# Silicon enhanced phosphorus uptake in rice under dry cultivation through root organic acid secretion and energy distribution in low phosphorus conditions

**DOI:** 10.3389/fpls.2025.1544893

**Published:** 2025-03-24

**Authors:** Hao Jiang, Wanchun Li, Zixian Jiang, Yunzhe Li, Xinru Shen, Min Nuo, Hongcheng Zhang, Bei Xue, Guangxin Zhao, Ping Tian, Meiying Yang, Zhihai Wu

**Affiliations:** ^1^ Faculty of Agronomy, Jilin Agricultural University, Changchun, Jilin, China; ^2^ Jilin Province Green and High Quality Japonica Rice Engineering Research Center, Jilin Agricultural University, Changchun, Jilin, China; ^3^ Changchun Farmers Vocational Education Center, Changchun Agriculture and Rural Bureau, Changchun, Jilin, China; ^4^ College of Life Sciences, Jilin Agricultural University, Changchun, Jilin, China

**Keywords:** phosphorus, silicon, rice, dry cultivation, root architecture, organic acid, microorganism, energy metabolism

## Abstract

Dry cultivation of rice (DCR) is one of the important rice cultivation practices aimed at addressing freshwater resource shortages. However, the non-renewable nature of phosphate resources constrains agricultural development. In the context of the contradiction between rice, water, and phosphorus, there is little research on using the silicon phosphorus relationship to improve the phosphorus availability and uptake of DCR. This experiment used field soil and established five fertilization treatments: no phosphorus application, low phosphorus and normal phosphorus (0, 25, 75 kg·ha^-1^ P_2_O_5_) (0P, 25P, 75P), along with two silicon levels (0, 45kg·ha^-1^ SiO_2_), resulting in the treatments 0P, 0PSi, 25P, 25PSi, and 75P. The soil phosphorus components and plant phosphorus uptake were analyzed. The results showed that adding silicon to 25P increased the Olsen-P content (14.37%) by increasing Ca_8_-P (9.04%) and Al-P (19.31%). Additionally, root and leaf phosphorus content increased by 7.6% and 5.8%, respectively, comparable to the levels observed in the 75P treatment. On one hand, adding silicon increases malate (40.48%) and succinate (49.73%) content, enhances acid phosphatase activity, and increases the abundance of *Bradyrhizobium*, *Paenibacillus*, and *Bacillus*, as well as the proportion of *Fusarium*, forming an “organic acid microbial” activated phosphorus system. On the other hand, the addition of silicon alleviated phosphorus limitations by reducing ATP consumption in roots through a decrease in ATPase and P-ATPase content. This also minimized excessive NSC transport to roots, thereby promoting shoot growth by downregulating *SUT1*, *SWEET11*, *SUS2*, and *CIN2*. In addition to optimizing root-to-shoot ratio and providing sufficient energy, silicon addition also increases root volume and upregulates *OsPT2*, *OsPT4*, and *OsPT8*, thereby promoting phosphorus uptake. In summary, 25PSi optimizes the root-to-shoot ratio and promotes phosphorus conversion and uptake through organic acid, microbial, and energy pathways. Applying silicon is beneficial for the sustainable and efficient management of phosphorus in DCR.

## Introduction

The International Food Policy Research Institute predicts that by 2050, climate change will cause a 20% decrease in irrigated rice production in developing countries, while available freshwater resources are expected to decline by 50% (Aditi et al., 2020). Insufficient moisture significantly impacts crop yields and threatens global food security (Maryam, 2021). Rice is a food crop with high water consumption, and addressing the “rice-water contradiction” is a pressing issue in rice-growing regions worldwide. In recent years, dry farming of rice has gradually become a research hotspot in the field of agricultural ecology. The ‘blue revolution’ concept for food security through water-saving and drought-resistant rice has been proposed, leading to the cultivation and widespread promotion of water-saving and drought-resistant rice varieties (Xia et al., 2022). Significant progress has been made in variety screening (Wei et al., 2022), quality assessment (Wang et al., 2023), and yield enhancement strategies (Jiang et al., 2023a) for rice cultivation under drought conditions. Numerous studies on the exploration of drought-resistant germplasm resources and drought-resistant mechanisms have demonstrated that dry rice cultivation has outstanding advantages, including water conservation, saving costs and labor, adapting to mechanization, and simplifying rice cultivation (Jiang et al., 2023b; Bi et al., 2021; Guo et al., 2024. As a result, it is currently a research hotspot in the field of water-saving cultivation of rice.

Phosphorus (P) is a key nutrient element for crop growth. Increased food production may drive the demand for phosphorus input in farmland, but phosphate ore is a non-renewable resource. The total amount of phosphorus fertilizer application in global farmland continues to increase, reaching 18 TgP yr^−1^ in 2013, which has exceeded the boundary value (6-12 TgP yr^−1^ to TgP yr^−1^). This figure is projected to increase to 22-27 TgP yr^−1^ by 2050 (Zou et al., 2022). Improper use of phosphorus in crop production leads to excessive leaching of phosphorus into water bodies, posing a threat to aquatic organisms and human health (Bouwman et al., 2017). In order to meet the growing demand for food, address the rice-water-phosphorus contradiction, and solve the multiple challenges of exacerbating phosphorus pollution and depleting phosphate rock reserves, it is essential to improve the phosphorus utilization efficiency in rice under dry cultivation production, particularly in low phosphorus environments.

There are various strategies plants use to improve soil phosphorus availability and uptake: (1) “P-mining”, which utilizes root exudates such as organic anions (carboxylates) and phosphatases to mineralize difficult to utilize inorganic and organic phosphorus pools (Wen et al., 2020); (2) Root foraging “, where plants alter their root system configuration to obtain more effective soil phosphorus (Liu, 2021); (3) Symbiotic relationships, such as root colonization with arbuscular mycorrhizal and ectomycorrhizal fungi (Song et al., 2021); (4) Metabolic adaptation, such as replacing phospholipids with non-phospholipids (such as galactolipids and thiolipids) (Hans et al., 2012); (5) Improved internal phosphorus utilization efficiency, which increases plant yield per unit of phosphorus uptake (Liao et al., 2020). We discovered that silicon can increase rice yield under dry cultivation under normal phosphorus conditions (Jiang et al., 2023a). However, the relationship between silicon and phosphorus under low phosphorus conditions remains to be explored, and the effects of silicon on the soil phosphorus environment and root phosphorus uptake are not yet clear.

The relationship between rhizosphere microbial communities and root exudates has become one of the research hotspots in root-soil interaction (Sreejata et al., 2024). However, the impact of silicon-phosphorus interaction in the rhizosphere of rice under dry cultivation on root exudates and microbial communities remains unclear. Phosphorus is well known to play a critical role in essential life processes such as respiration and energy metabolism. Plant metabolic activities, ion transport, ineffective cycling, sucrose transport, and response to abiotic stress require a significant amount of energy (Jeffrey et al., 2019). In cases of phosphorus deficiency, the root-to-shoot ratio of crops often increases (Xiao et al., 2022; Hermans et al., 2006). However, the role of energy metabolism in root development of rice under dry cultivation induced by phosphorus scarcity remains unclear. Therefore, we propose two scientific questions (1) What is the root-soil microbial relationship of silicon-phosphorus interaction under dry cultivation? (2) How does silicon regulate phosphorus absorption and development through root energy metabolism in response to changes in phosphorus environment? This experiment aims to develop a “water-saving, phosphorus-reducing, and efficiency-enhancing” technology model for rice while also elucidating the root-soil-microbial interaction mechanism of silicon phosphorus interaction. This will provide new solutions to address the rice-water-phosphorus contradiction.

## Materials and methods

### Site description

The experiment was conducted from 2023 to 2024 at the National Crop Variety Approval Characteristic Appraisal Station (125°39 E, 44°46 N) of Jilin Agricultural University in Changchun, Jilin Province, China. The 0-20 cm soil layer has a clay loam structure with a pH value of 6.3, organic matter of 16.5 g·kg^-1^, available silicon of 96.45 mg·kg^-1^, alkaline nitrogen of 92.3 mg·kg^-1^, available phosphorus of 9.6 mg·kg^-1^, and available potassium of 208.46 mg·kg^-1^. Over the past three years, the accumulated temperature and rainfall during the entire growth period in the region were 2873°C and 674.6 mm, respectively.

### Experimental design and crop management

This experiment uses the rice variety ‘Suigeng 18’ (China Rice Data Center, No. 2014021, https://www.ricedata.cn/variety/varis/614593.htm) as the material. Research indicates that ‘Suigeng 18’ is a rice variety suitable for dry farming in the central region of Jilin Province (Jiang et al., 2021; Wei et al., 2022).

Potted experiments were conducted in 2023 and 2024, while field experiments were conducted in 2024. This experiment included three levels of phosphorus (P_2_O_5_) fertilizer (0 kg·ha^-1^、25 kg·ha^-1^、75 kg·ha^-1^) and two levels of silicon (SiO_2_) fertilizer (0 kg·ha^-1^、45 kg·ha^-1^), resulting in five treatments: 0P, 0PSi, 25P, 25PSi, and 75P. The soil for the pot experiment was sourced from the field, with 8.5 kg of soil used per pot. Three holes were sown per pot, with eight seeds per hole. Each treatment was repeated three times. The field experimental plot covers an area of 25 m^2^ and is sown using the dry broadcasting method with a seeding rate of 150 kg·ha^-1^. The strip sowing method is used with a row spacing of 30 cm, and each treatment is repeated three times.

Silicon, phosphorus, and potassium fertilizers are applied simultaneously as base fertilizers. The silica fertilizer is derived from Russian mineral silica (Biotronik, Berlin, Germany), with an effective silica content of ≥ 72% (**Supporting Information**, **Supplementary Figure S1**). The phosphate fertilizer is composed of superphosphate (P_2_O_5_ 12%). The potassium fertilizer consists of 75 kg·ha^-1^ potassium chloride (K_2_O 60%). The nitrogen fertilizer consists of urea (pure N 46%) applied at a rate of 160 kg·ha^-1^, with a distribution ratio of 5:3:2 for base fertilizer, tiller fertilizer, and spike fertilizer. The entire growth period is mainly rain-fed, and a soil water potential analyzer (SYS-TSS1, Sayas Technology Co., Ltd, Liaoning, China) is used to monitor changes in water potential in the experimental field. When the soil water potential at a depth of 10-15 cm falls below -35 kPa, a fixed spray 360° atomizing rotary sprinkler is used, with a spraying radius of 8 m and a water output of 0.7 m^3^·h^-1^. After replenishment, the soil water potential reaches -10 kPa. Water control in the potted plant experiment is consistent with that in the field experiment. Other measures are implemented according to the requirements for high-yield cultivation.

### Plant and soil sampling

45 days after sowing, the rhizosphere soil attached to the surface of the root system was collected, visible plant materials were removed, and the soil was sieved (2 mm). The soil was then analyzed for microorganisms, acid phosphatase activity, available phosphorus content, and inorganic phosphorus grading. Fresh root systems were collected for quantitative analysis of root architecture, carboxylates, energy metabolism, and gene fluorescence. Biomass, root phosphorus content, and non-structural carbohydrates were measured after separating plant roots, stems, and leaves.

### Morphological indicators and plant biomass

#### Root system architecture

Root images were scanned using a root morphology scanner (Epson Perfection V800 photo; EPSON, Tokyo, Japan) and stored on a computer. Root length, root surface area, and root volume were analyzed using the root analysis system software WinRhizo PRO 2016 (Regent Instruments, Quebec, Canada).

#### Leaf area and biomass

Leaf area was measured using a leaf area analyzer (CID-203; CID Bioscience, Camas, WA, USA). The plant was decomposed into three parts: roots, stems, and leaves, and placed in sulfuric acid paper bags. This process was repeated three times. The drying process was carried out at 105 °C for 30 minutes, then at 80 °C until constant weight was reached. The weight was recorded. Root/shoot ratio=root weight/stem and leaf weight.

### Plant phosphorus content and physicochemical properties

#### Plant phosphorus content

The dried samples were crushed by a powder machine and mixed well. 0.05 g of the sample was weighed and placed in a digestion tube, and then mixed and boiled with H_2_SO_4_-H_2_O_2_. Repeat three times. After cooling, adjust the volume and determine the total phosphorus content using the molybdenum antimony colorimetric method (Siddiqi and Glass, 1981).

#### Non-structural carbohydrates

The soluble sugar and starch content were determined using an improved sulfuric acid anthrone colorimetric method (DuBois et al., 2002). The content of total non-structural carbohydrates (NSC) was calculated as the sum of soluble sugars and starch content. NSC root/shoot ratio= (NSC×root weight)/(NSC×shoot weight).

### Root energy metabolism

The content of ATP, ATPase, plasma membrane H^+^-ATPase (P-ATPase), V-ATPase, alternative oxidase (AOX) was measured. Frozen roots (0.01g) were ground into fine powder in liquid nitrogen, homogenized in 0.01M PBS (pH 7.2), and centrifuged at 2000g for 20 minutes. The content of ATP, ATPase, P-ATPase, as well as the activity of V-ATPase and AOX were measured using the supernatant, following the manufacturer’s instructions (Shanghai Enzyme‐Linked Biotechnology Co., Ltd.).

Measurements of mitochondrial complex activities. Frozen root systems (0.01g) were ground and mitochondria were extracted in an ice bath using reagents from the mitochondrial complex activity assay kit. The extract underwent 30 cycles of ultrasound treatment (power, 20%; ultrasound time, 3s; interval time, 10s). The activity of complex I and complex V was measured according to the manufacturer’s instructions (Comin Biotechnology Co., Ltd.) using a mitochondrial complex activity assay kit (Li et al., 2023).

### Root gene expression level

The root system was frozen with liquid nitrogen and transferred to a -80°C freezer. After grinding the tissue in liquid nitrogen, total RNA was extracted using TriPure reagent (Aidlab Biotechnologies). RNA was reverse transcribed into single-stranded cDNA using ReverTra Ace qPCR RT Master Mix (TOYOBO). Real-time fluorescent quantitative PCR amplification was carried out using cDNA as a template. The reaction system consisted of a 20 μL reaction system, 10 μL of 2×SuperReal PreMix Plus, 8 μL ddH_2_O, 0.5 μL of upstream and downstream primers, and 1 μL of cDNA template. The PCR amplification reaction system was pre-denatured at 95°C for 15 minutes. The following 40 cycles were performed: pre-denaturation at 95°C for 20 seconds, annealing at 60 °C for 20 seconds, extension at 72 °C for 20 seconds, and melting for 6 seconds. Relative gene expression was analyzed using the 2^–ΔΔCT^ method. Primer information is shown in **Supplementary Table S1**.

### Collection of root exudates and soil analysis

#### Soil available phosphorus and inorganic phosphorus components

The crop roots were excavated, and the “shaking soil method” was used to remove residual non-rhizosphere soil from the roots while retaining rhizosphere soil. The rhizosphere soil was sealed in plastic bags, transported to the laboratory for air drying, and stored at room temperature (25°C). Effective phosphorus was extracted using 0.5 mol·L^-1^ NaHCO_3_ (Olsen et al., 1954) and measured by spectrophotometry at 700 nm (T3202, Youke Instrument, Shanghai, China). Six types of soil inorganic phosphorus components were extracted from soil samples using a sequential extraction program, including dicalcium phosphate (Ca_2_-P), octacalcium phosphate (Ca_8_-P), aluminum phosphate (Al-P), iron phosphate (Fe-P), occluded phosphate (O-P) and apatite (Ca_10_-P). The measurement of inorganic phosphorus components follows the method of Liu et al (Liu et al., 2022).

#### Root exudates

The roots and attached rhizosphere soil were soaked in a beaker containing 100 mL of CaCl_2_ solution (0.2 μmol·L^-1^) (Yu et al., 2023). After soaking the roots for 60 seconds, the root sheath soil was removed as much as possible to reduce root damage. 10 ml of supernatant was transferred to a centrifuge tube containing 2 drops of microbial inhibitor and 3 drops of concentrated phosphoric acid. The sample was stored at 20 °C until it passed through a 0.22 μm filter and analyzed for tartrate, succinate, malate, and citrate content by HPLC-MS/MS. The measurement of carboxylate salts follows the method of Fiori et al. (2018). The remaining soil in the beaker was dried and weighed to calculate the concentration of carboxylate based on the weight of the root sheath soil.

#### Soil acid phosphatase and microorganisms

A portion of the rhizosphere soil was stored at 4°C for acid phosphatase measurement, while another portion was stored at -80°C for microbial analysis. Soil acid phosphatase was determined using the colorimetric method with sodium phenylene phosphate.

Genomic DNA of microbial communities was extracted from soil samples using the magnetic bead soil DNA assay kit (RT 405-02; TIANGEN, Beijing, China). DNA extract was detected by 2% agarose gel electrophoresis, and DNA concentration and purity were determined by NanoDrop 2000 (Thermo Fisher Scientific, Waltham, MA, USA). Using a 10 ng DNA template, 0.2 µM forward and reverse primers, and 15 µL Phusion ^®^ High fidelity PCR Master Mix (New England Biolabs, Ipswich, MA, USA), the highly variable region ITS 1 of the fungal ITS rRNA gene was amplified. The samples were processed on the NovaSeq 6000 platform (Novogene Technology Co, Ltd., Tianjin, China) and clustered into operational taxonomic units (OTUs) at the 97% threshold. The DADA2 module in QIIME2 software was used to denoise and filter out sequences with an abundance less than 5, yielding the final amplicon sequence variations (ASVs) and feature table.

### Statistical analysis

Microsoft Excel 2021 (Microsoft, Redmond, United States) was used to organize the data. All experiments were conducted in triplicate (n = 3). One-way analysis of variance (ANOVA) and statistical analysis were performed to determine statistically significant differences between the control and other treatments using SPSS 27 software (SPSS, Chicago, United States). At *P*<0.05, the difference was considered statistically significant. Origin 2022 software (OriginLab, Northampton, USA) was used to generate figures.

## Results

### Soil phosphorus components and root phosphorus uptake under different silicon and phosphorus conditions

**Figure 1:**
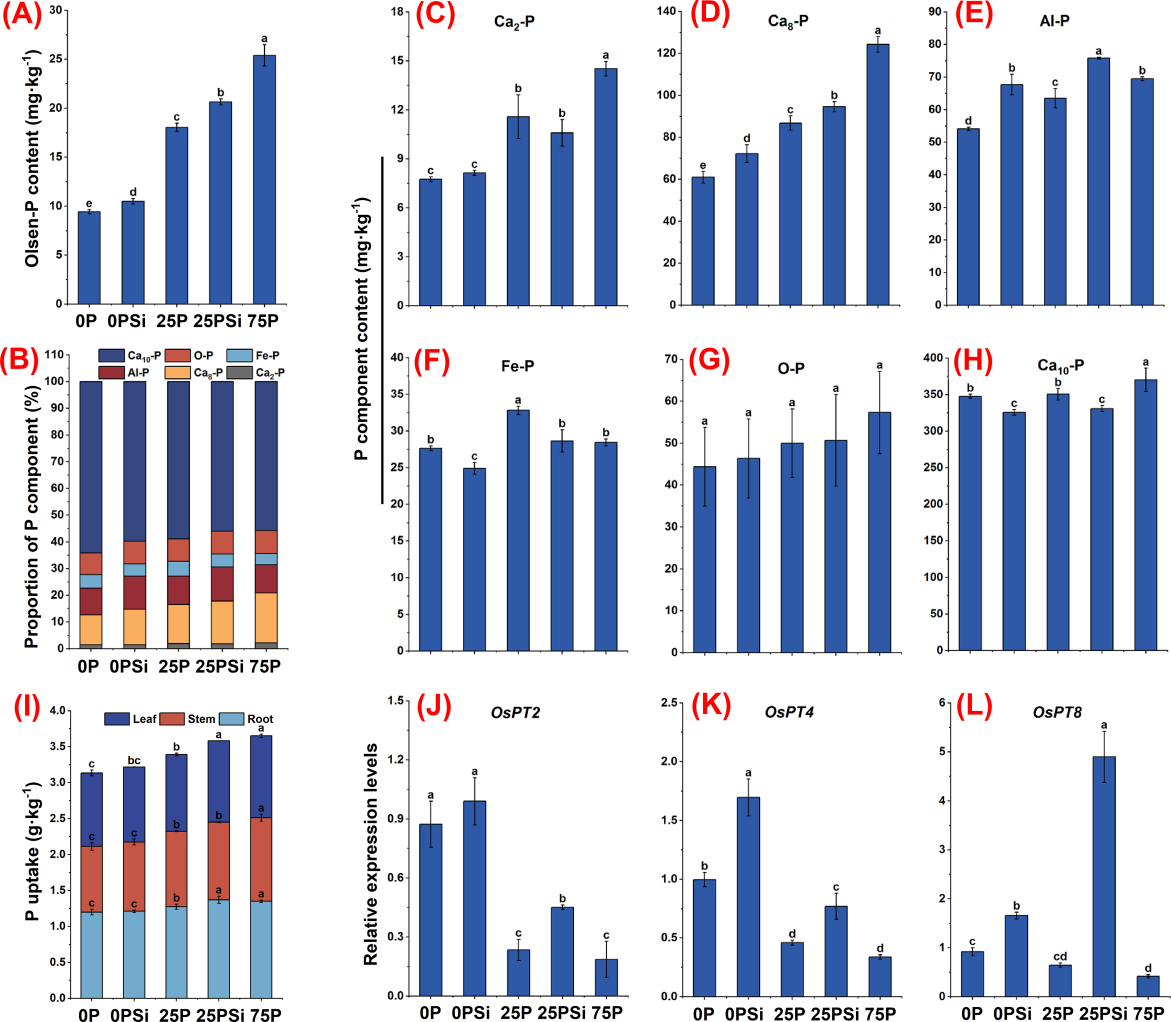
The effect of silicon and phosphorus on the phosphorus environment and root phosphorus uptake in rice under dry cultivation. **(A)**, Olsen-P content. **(B)**, Proportion of phosphorus (P) components. **(C–H)**, Phosphorus component content, **(C)**, Ca_2_^-^P; **(D)**, Ca_8_^-^P; **(E)**, Al-P; **(F)**, Fe-P; **(G)**, O-P; **(H)**, Ca_10_^-^P. **(I)**, Plant phosphorus uptake. **(J–L)**, Relative expression levels, **(J)**, *OsPT2*; **(K)**, *OsPT4*; **(L)**, *OsPT8*. Different letters represent significant differences in the same indicator between different treatments (*P*<0.05).

Regarding the soil phosphorus environment, the addition of silicon significantly increased the Olsen-P content in the 0P and 25P treatments (*P<0.05*) (**Figure 1A**), with increases of 11.36% and 14.37%, respectively. In terms of the proportion of phosphorus components. Adding silicon increased the proportion of circulating phosphorus components (Ca_8_-P, Al-P), which were 17.98% and 24.58% higher than the 0P treatment and 9.75% and 20.09% higher than the 25P treatment. Silicon addition reduces the proportion of refractory phosphorus component (Ca_10_-P), which is less than 0P and 25P, respectively, by 6.78% and 4.94% (**Figure 1B**). In terms of different phosphorus component contents. The easily absorbable Ca_2_-P content showed a significant increase trend (*P<0.05*) among different phosphorus dosages (**Figure 1C**). The addition of silicon significantly increased the content of Ca_8_-P and Al-P components in the medium cycle phosphorus treated with 0P and 25P (*P<0.05*), while the addition of silicon under the 25P treatment increased Ca8-P and Al-P contents by 9.04% and 19.31%, respectively (**Figures 1D, E**). Conversely, silicon addition significantly reduced the Fe-P content (*P<0.05*). However, no significant difference was observed between 25PSi and 75P (*P>0.05*) (**Figure 1F**). The content of refractory phosphorus components O-P and Ca_10_-P, was highest in the 75P treatment. O-P exhibited an increasing trend across the treatments, but there was no significant difference (*P>0.05*) (**Figure 1G**). The addition of silicon significantly reduced the Ca_10_-P content in 0P and 25P treatments by 6.34% and 5.55%, respectively (*P<0.05*) (**Figure 1H**). It is evident that silicon addition increases the proportion of circulating phosphorus components, reduces Ca_10_-P content, and increases the soil Olsen-P content.

Silicon addition significantly increased phosphorus content in the roots and leaves of 25P by 7.6% and 5.8%, respectively (*P<0.05*) (**Figure 1I**), but there was no significant difference between 25PSi and 75P (*P>0.05*). From the overall perspective of roots, stems, and leaves, adding silicon increased the phosphorus content of 0P and 25P plants by 2.65% and 5.58%, respectively. Silicon addition significantly increased the expression levels of phosphorus transport genes *OsPT4* and *OsPT8* in the 0P and 25P treatments (**Figures 1K, L**). There was no significant change in the expression level of *OsPT2* with silicon addition at 0P; however, silicon addition at 25P significantly upregulated the expression level of *OsPT2* (**Figure 1J**). It is evident that silicon addition upregulates the expression levels of phosphorus transporters in 0P and 25P treatments, significantly increasing the phosphorus content in 25P.

### Plant growth and RSA under different silicon and phosphorus conditions

**Figure 2:**
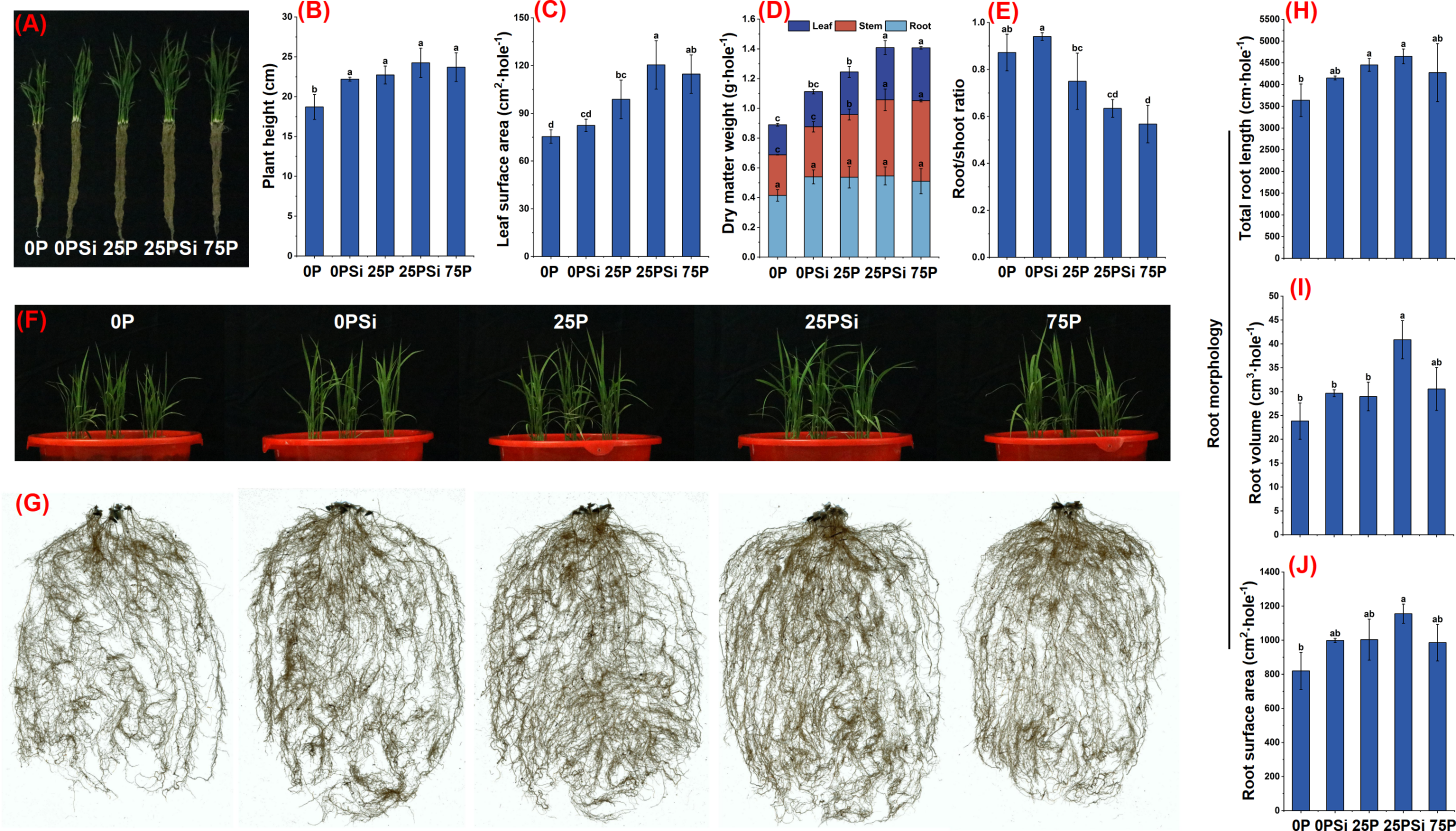
Effects of silicon and phosphorus on plant growth and RSA of rice under dry cultivation. **(A)**, Photos of plants; **(B)**, plant height; **(C)**, leaf surface area; **(D)**, dry matter weight; **(E)**, root/shoot ratio; **(F)**, photos of aboveground parts of plants; **(G)**, root photo. **(H–J)**, Root morphology with different diameters, **(H)**, root length; **(I)**, root volume; **(J)**, root surface area. Different letters represent significant differences in the same indicator between different treatments (*P*<0.05).

The plant height was significantly lower in the 0P treatment than in other treatments, and reached its highest at 25PSi (**Figures 2A, B**). The addition of silicon increased the leaf area in the 0P and 25P treatments by 9.31% (*P>0.05*) and 21.93% (*P<0.05*), respectively, with no significant difference between 25PSi and 75P (**Figures 2C, F**). The biomass of various parts of the plant increased after silicon addition (**Figure 2D**). There was no significant difference in root weight among the treatments (**Figure 2G**). However, there was a significant difference in stem and leaf weight between 25P and 25PSi *(P<0.05*), while no significant difference was observed between 25PSi and 75P (*P>0.05*). Compared with 0P, the root shoot ratio of 25PSi decreased by 27.29%, and decreased by 15.43% compared to 25P (**Figure 2E**). After adding silicon, the total root length, volume, and surface area of 0P and 25P treatments increased (**Figures 2H-J**), with silicon significantly increasing the root volume of 25P treatment *(P<0.05*) (**Figure 2I**). It is evident that silicon addition increased plant height, leaf area, and biomass of roots, stems, and leaves in the 0P and 25P treatments, with the 25PSi treatment showing the highest levels. The promotion effect of silicon on aboveground growth is accompanied by a decrease in root-to-shoot ratio. The two-year pot experiment and field experiment showed the same trend (**Supporting Information**, **Supplementary Figures S2**, **S3**).

### Carboxylate secretion by roots under different silicon and phosphorus conditions

**Figure 3:**
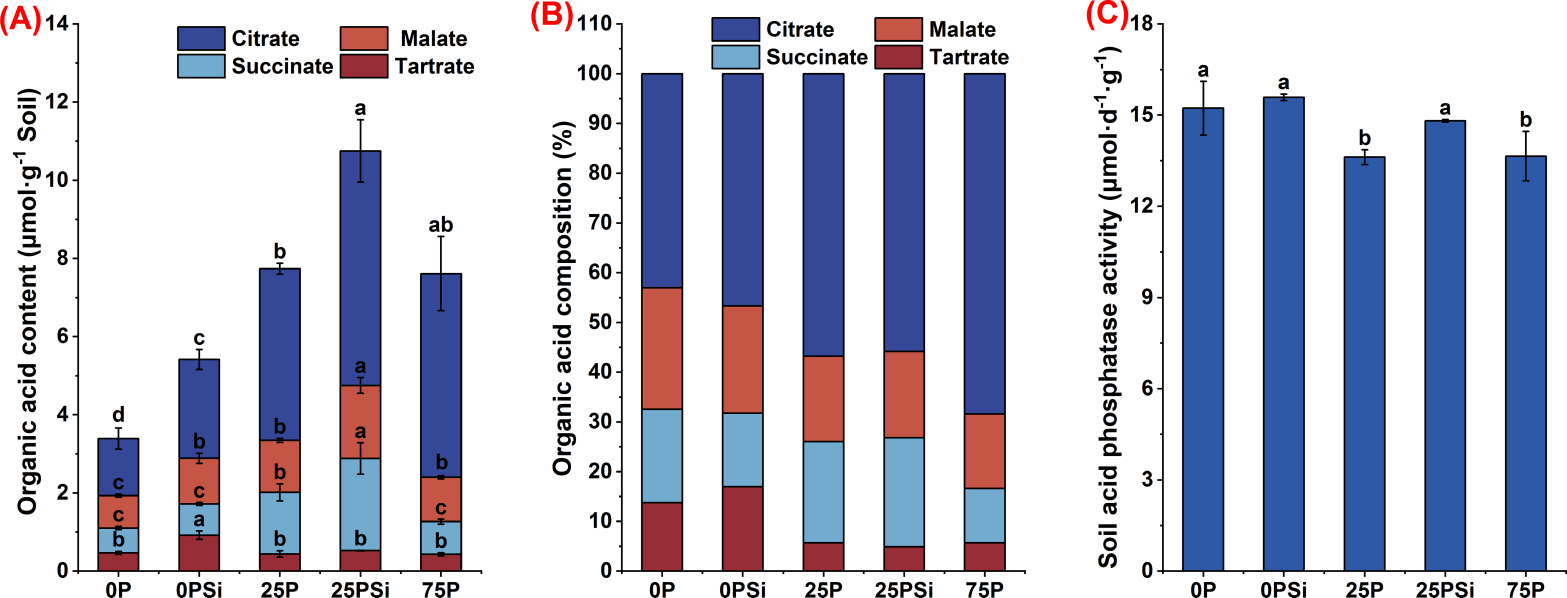
Effects of silicon and phosphorus on carboxylate secretion by the rice root system under dry cultivation and soil acid phosphatase activity. **(A)**, Carboxylates content. **(B)**, Carboxylate composition. **(C)**, Activity of soil acid phosphatase. Different letters represent significant differences in the same indicator between different treatments (*P*<0.05).

Silicon addition promotes carboxylate secretion, with the root system treated with 25PSi exhibiting the highest carboxylate secretion content (**Figure 3A**). Silicon addition significantly promoted the content of malate and citrate in 0P and 25P treatments (*P<0.05*). Malate content increased by 40.98% and 40.48%, while citrate content increased by 73.26% and 36.52%, respectively, with the 25PSi treatment showing higher values than the 75P treatment. Additionally, silicon addition significantly increased the tartrate content in the 0P treatment (*P<0.05*) and significantly promoted the succinate content in the 25P treatment (P<0.05). The tartrate content in the 0PSI treatment and the succinate content in the 25PSi treatment were significantly higher than those in other treatments (*P<0.05*). Across all treatments, citrate accounted for the highest proportion, while tartrate had the lowest proportion (**Figure 3B**). These findings indicate that the 25PSi treatment resulted in the highest carboxylate secretion. Silicon addition primarily increased the content and proportion of citrate and tartrate in the 0P treatment, while enhancing the content and proportion of malate and succinate in the 25P treatment.

Soil acid phosphatase activity exhibited a decreasing trend among different phosphorus dosages. Silicon addition increased enzyme activity in 0P and 25P treatments by 2.38% (*P>0.05*) and 8.79% (*P<0.05*), respectively (**Figure 3C**).

### Soil microbial diversity under different silicon and phosphorus conditions

**Figure 4:**
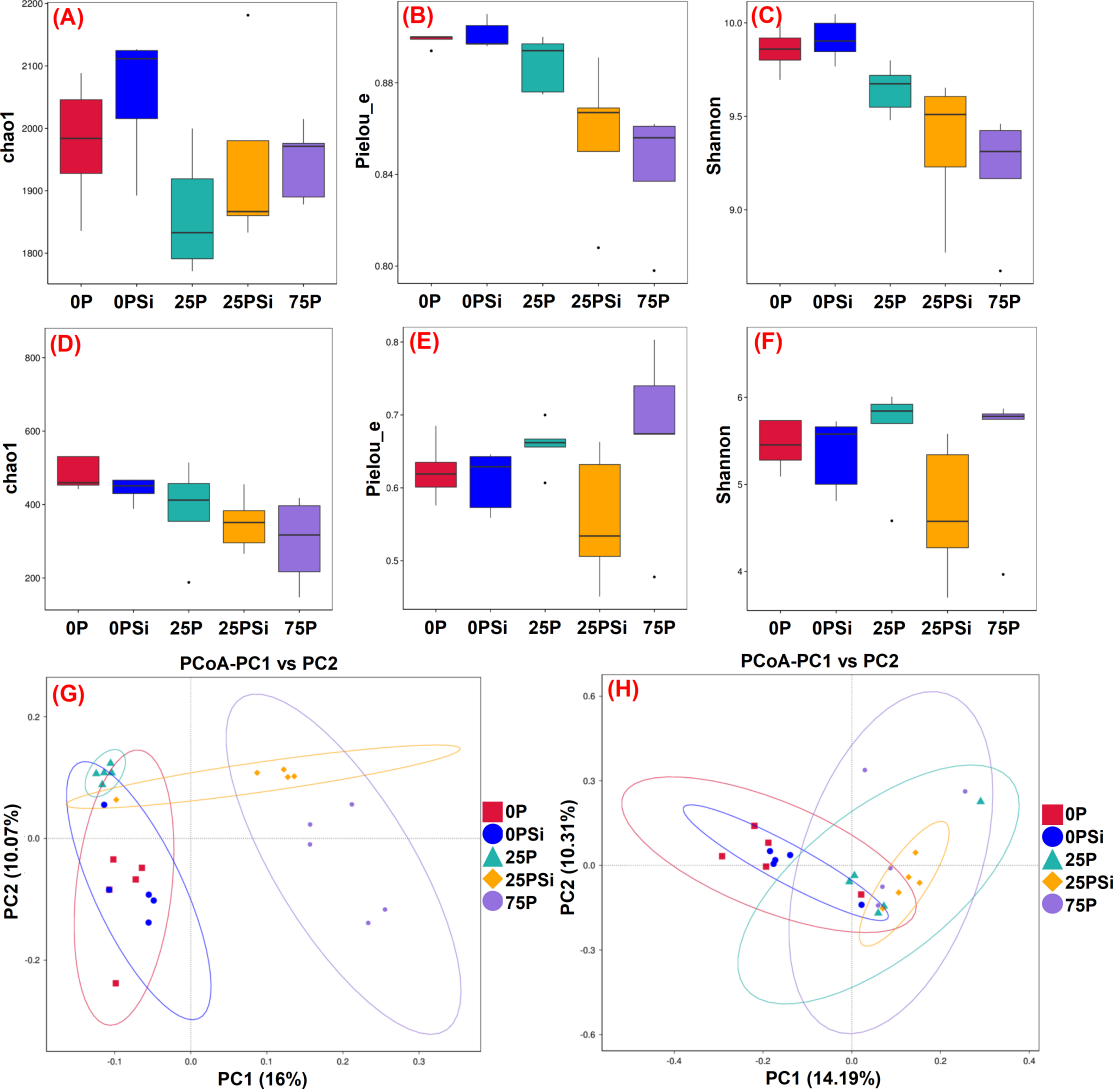
Effects of silicon and phosphorus on the α-diversity and β-diversity of soil microorganisms in rice under dry cultivation. **(A–C)** and **(D-F)**, α-diversity of bacteria and fungi, **(A, D)**, chao1; **(B, E)**, pielou_e; **(C, F)**, shannon. **(G, H)**, β-diversity of bacteria and fungi.

This experiment uses 16S rRNA and ITS rRNA to examine changes in microbial communities. The larger the chao1 index, the more low-abundance species there are in the community. Similarly, the larger the pielou-e index and shannon index, the more evenly distributed the species. For bacterial communities, the addition of silicon increased the chao1 indices in the 0P and 25P treatments (**Figure 4A**). The pielou-e and shannon indices were similar between the 0P and 0PSi treatments, but showed a successive decline in the 25P, 25PSi, and 75P treatments (**Figures 4B, C**). PCoA analysis revealed that PC1 explained 16% of the variation, while PC2 explained 10.07% of the variation (**Figure 4G**). These results indicates differences in bacterial composition in soil after silicon addition. Furthermore, compared with the 25P treatment, the bacterial community composition in the 25PSi treatment was more extensive.

For fungal communities, silicon addition reduced the chao1 indices in the 0P and 25P treatments, with the 75P treatment showing the lowest values (**Figure 4D**). The addition of silicon significantly decreased the pielou-e and shannon index of the 25P treatment by 15.37% and 16.33%, respectively (**Figures 4E, F**). PC1 accounted for 14.19% of the fungal community variation in the sample, while PC2 accounted for 10.31% of the fungal community variation in the sample (**Figure 4H**). Significant group segregation was observed under different phosphorus dosages. Compared with the 0P treatment, the soil fungal composition in the 25P, 25PSi, and 75P treatments differed, leading to distinct fungal community structures in the soil. These findings suggest that silicon addition increases the number of bacterial species, whereas the combination of silicon and 25P reduces fungal diversity.

### Soil microbial community structure and composition under different silicon and phosphorus conditions

**Figure 5:**
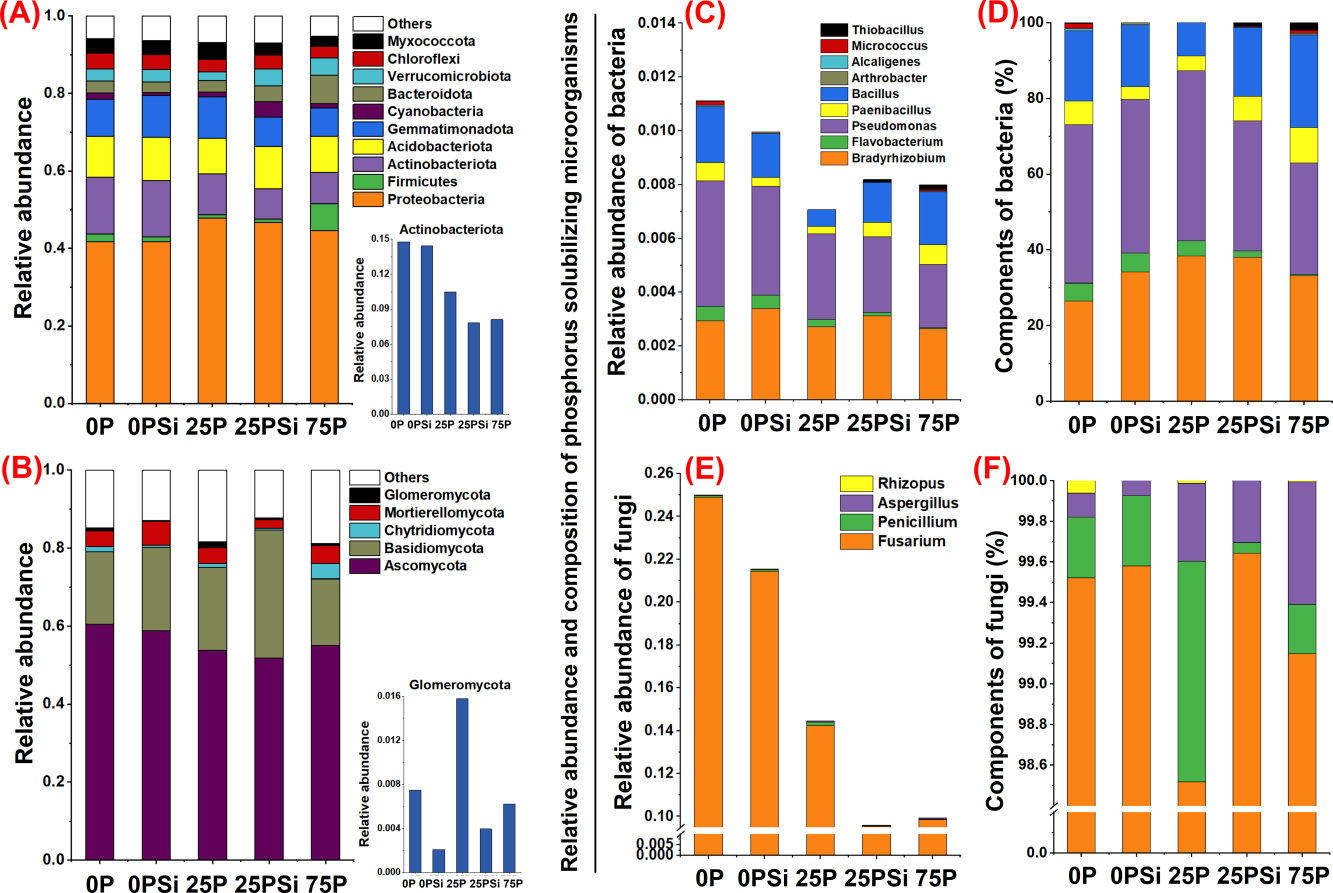
Effects of silicon and phosphorus on the abundance of soil microbial phyla and phosphorus solubilizing microbial genera in rice under dry cultivation. **(A, B)**, Relative abundance of bacteria and fungi. **(C–F)**, Relative abundance and composition of phosphorus solubilizing microorganisms, **(C, D)**, phosphate solubilizing bacteria; **(E, F)**, phosphate solubilizing fungi.

The abundance of bacterial communities at the phylum level is shown in **Figure 5A**. The top 10 most abundant phyla across all samples are *Myxococcota*、*Chloroflexi*、*Verrucomicrobiota*、*Bacteroidota*、*Cyanobacteria*、*Gemmatimonadota*、*Acidobacteriota*、*Actinobacteriota*、*Firmicutes* and *Proteobacteria*. Silicon addition promotes the abundance of *Acidobacteriota* and *Verrucomicrobota* under 0P and 25P treatments. Silicon addition promotes the abundance of *Gemmatimonadota* and *Myxococcata* under 0P conditions and increases the abundance of *Cyanobacteria*, *Bacteroidota*, and *Chloroflexi* treated under 25P conditions. Conversely, the abundance of *Actinobacteriota*, which is associated with phosphorus solubilization, showed a decreasing trend after silicon addition.

The abundance of fungal communities at the phylum level is shown in **Figure 5B**. *Glomeromycota*、*Mortierellomycota*、*Chytridiomycota*、*Basidiomycota* and *Ascomycota* are the top 5 most abundant phyla among all samples. Adding silicon increases the abundance of *Basidiomycota* by 0P and 25P treatments, and also increases the abundance of *Mortierellomycota* by 0P treatment. The abundance of *Glomeromycota* associated with arbuscular mycorrhizal fungi (AMF) showed a decreasing trend after adding silicon addition.

Thirteen phosphorus solubilizing microorganisms were identified, including nine phosphorus solubilizing bacteria (**Figure 5C**) and four phosphorus solubilizing fungi (**Figure 5E**). In terms of bacteria, silicon addition promoted the abundance of *Bradyrhizobium* under 0P and 25P treatments, while reducing the abundance of *Flavobacteria* and *Pseudomonas*. Among most phosphorus-solubilizing bacteria, it was found that silicon addition also increased the abundance and proportion of *Paenibacillus* and Bacillus under 25P treatment. Additionally, it increased the abundance and proportion of *Arthrobacter* under 0P and *Microcystis* and *Thiobacillus* under 25P, although these three bacteria accounted for a relatively small proportion of the phosphate solubilizing bacteria (**Figures 5C, D**). In terms of fungi, silicon addition reduced fungal abundance under 0P and 25P treatments, increased the proportion of *Fusarium* and *Penicillium* under 0P treatment, and increased the proportion of *Fusarium* under 25P treatment (**Figures 5E, F**). In summary, silicon addition induced distinct microbial changes under 0P and 25P treatments, notably decreasing *Actinobacteriota* and *Glomeromycota* abundance at the phylum level. The specific recruitment effect of silicon on phosphate-solubilizing bacteria is primarily reflected in the increased abundance of *Bradyrhizobium* and the proportion of fungi *Fusarium* and *Penicillium* under 0P treatment, and in the increased abundance of *Bradyrhizobium*, *Paenibacillus*, and *Bacillus*, along with the proportion of fungi *Fusarium* under 25P treatment.

### Root sugar transport and metabolism under different silicon and phosphorus conditions

**Figure 6:**
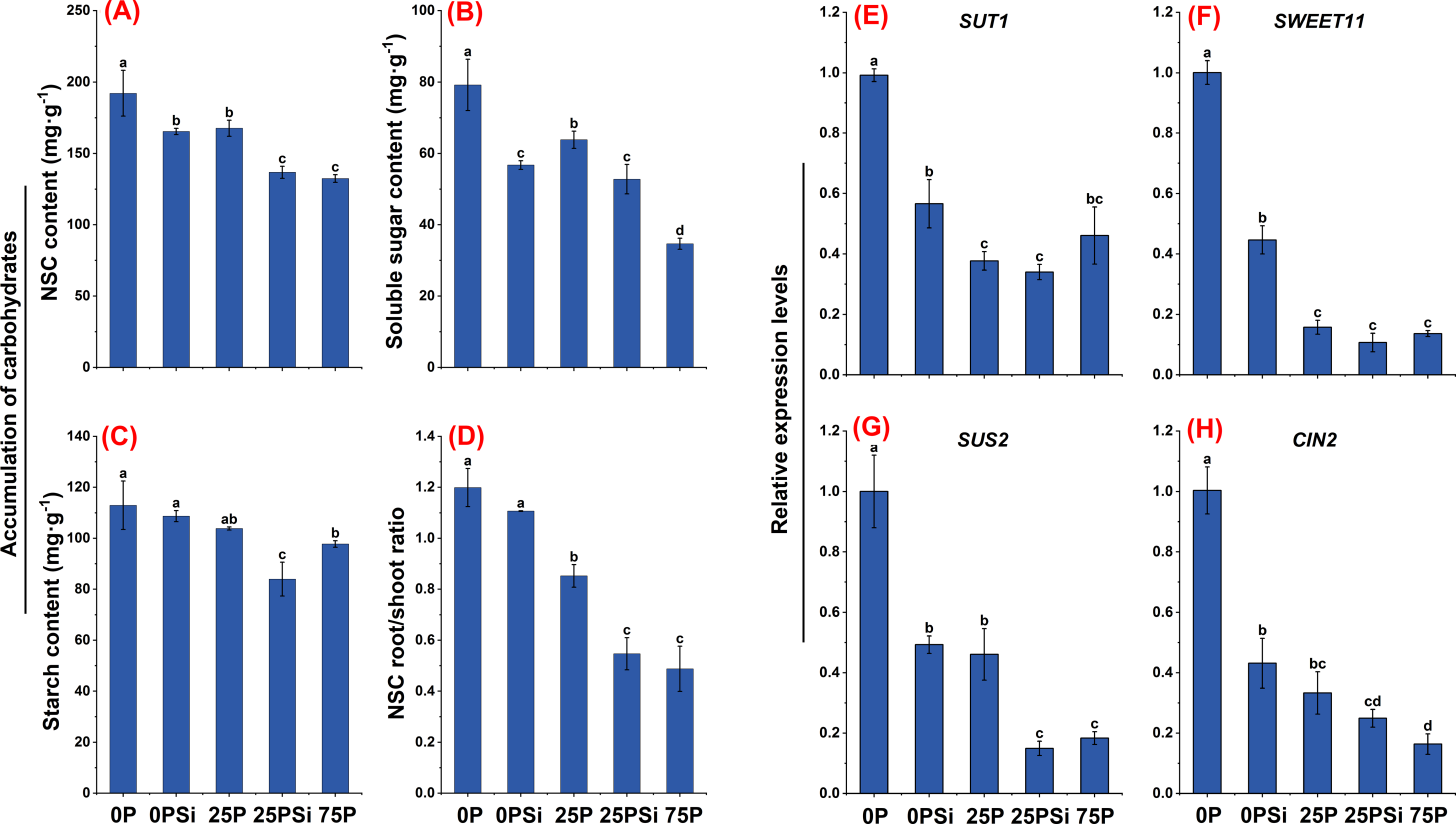
Effects of silicon and phosphorus on root sugar transport and metabolism in rice under dry cultivation. **(A–D)**, Accumulation of carbohydrates, **(A)**, non-structural carbohydrate (NSC) content; **(B)**, soluble sugar content; **(C)**, starch content; **(D)**, NSC root/shoot ratio. **(E–H)**, Relative expression levels, **(E)**, *SUT1*; **(F)**, *SWEET11*; **(G)**, *SUS2*; **(H)**, *CIN2*. Different letters represent significant differences in the same indicator between different treatments (*P*<0.05).

As the phosphorus dosage increased, starch, soluble sugar, and NSC showed a decreasing trend. NSC and soluble sugar showed significant differences among different phosphorus dosages (*P<0.05*). Silicon addition significantly reduced the soluble sugar content in the 0P treatment and the starch and soluble sugar content in the 25P treatment (*P<0.05*) (**Figures 6B, C**). Silicon addition significantly reduced the NSC content in both the 0P and 25P treatments (*P<0.05*) (**Figure 6A**), and decreased the NSC root-to-shoot ratio (**Figure 6D**). However, there was no significant difference in the root-to-shoot ratio between 25PSi and 75P treatments (*P>0.05*).

SUTs and SWEETs are involved in the allocation of assimilates in plants, and several genes involved in sucrose transport were studied. Silicon addition downregulated the expression levels of *SUT1* and *SWEET11* in both the 0P and 25P treatments, reaching significant levels between 0P and 0PSi (*P<0.05*). No significant difference was found between 25PSi and 75P (*P>0.05*) (**Figures 6E, F**). SUS and CIN play key roles in sucrose metabolism. The expression levels of *SUS2* and *CIN2* were downregulated with increasing phosphorus and silicon addition. Silicon addition significantly downregulated the expression levels of *SUS2* and *CIN2* in the 0P treatment (*P<0.05*), and significantly reduced the expression level of *SUS2* in the 25P treatment (*P<0.05*). There was no significant difference between 25PSi and 75P (*P>0.05*) (**Figures 6G, H**). In summary, compared to 75P, 0P and 25P allocate more NSC to the roots, while silicon addition reduces the allocation of sugar to the roots.

### Root energy metabolism under different silicon and phosphorus conditions

**Figure 7:**
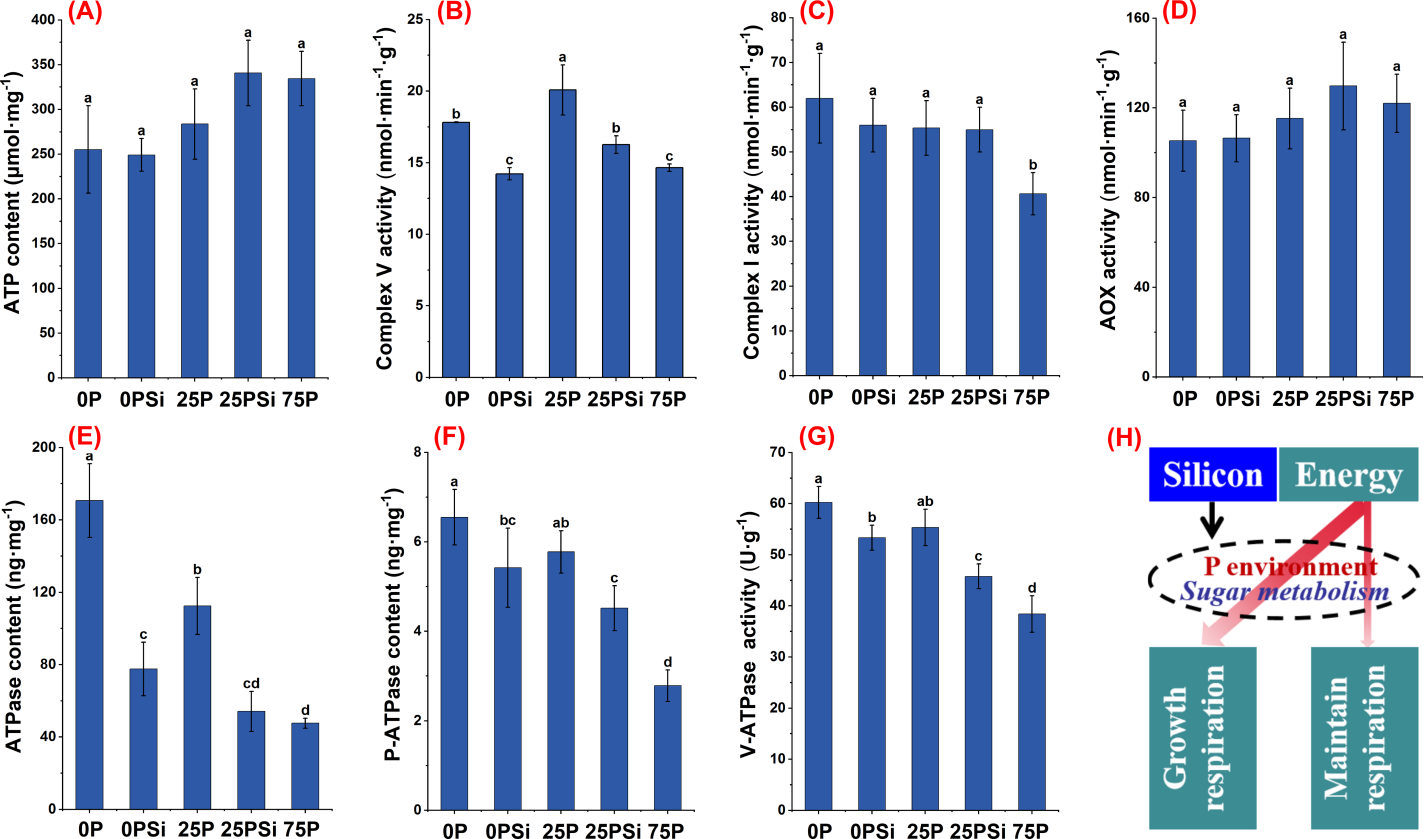
Effects of silicon and phosphorus on energy metabolism of rice under dry cultivation. **(A)**, ATP content; **(B)**, Complex V activity; **(C)**, Complex I activity; **(D)**, Alternative oxidase (AOX) activity; **(E)**, ATPase content; **(F)**, Plasma membrane H^+^-ATPase (P-ATPase) content; **(G)**, V-ATPase activity; **(H)**, Schematic diagram of silicon affecting energy distribution. Different letters represent significant differences in the same indicator between different treatments (*P*<0.05).

We investigated the mitochondrial respiratory electron transport chain complex to elucidate the role of silicon in affecting root energy production. Silicon addition increased the ATP content in the 25P treatment, which was similar to the 75P treatment (**Figure 7A**). The activity of complex V showed significant differences among different phosphorus dosages (*P<0.05*). The addition of silicon significantly reduced the activity of complex V in the treatments with 0P and 25P (**Figure 7B**). The activity of complex I was highest in the 0P treatment and lowest in the 75P treatment, with no significant change after silicon addition (**Figure 7C**). AOX is an important factor influencing the energy production efficiency in plants. Silicon addition increased the AOX activity in the 0P and 25P treatments, although the difference was not significant (*P>0.05*) (**Figure 7D**).

In terms of energy utilization, the content of ATPase, P-ATPase, and V-ATPase activity decreased with increasing phosphorus dosage and silicon addition. Silicon addition significantly reduced ATPase, P-ATPase content, and V-ATPase activity in 0P and 25P treatments (**Figures 7E-G**). In summary, compared to the 75P treatment, the 0P and 25P treatments promote energy generation and increase energy consumption, while adding silicon reduces energy consumption (**Figure 7H**).

## Discussion

### The effect of silicon addition on plant growth and RSA under low phosphorus conditions in rice under dry cultivation

**Figure 8:**
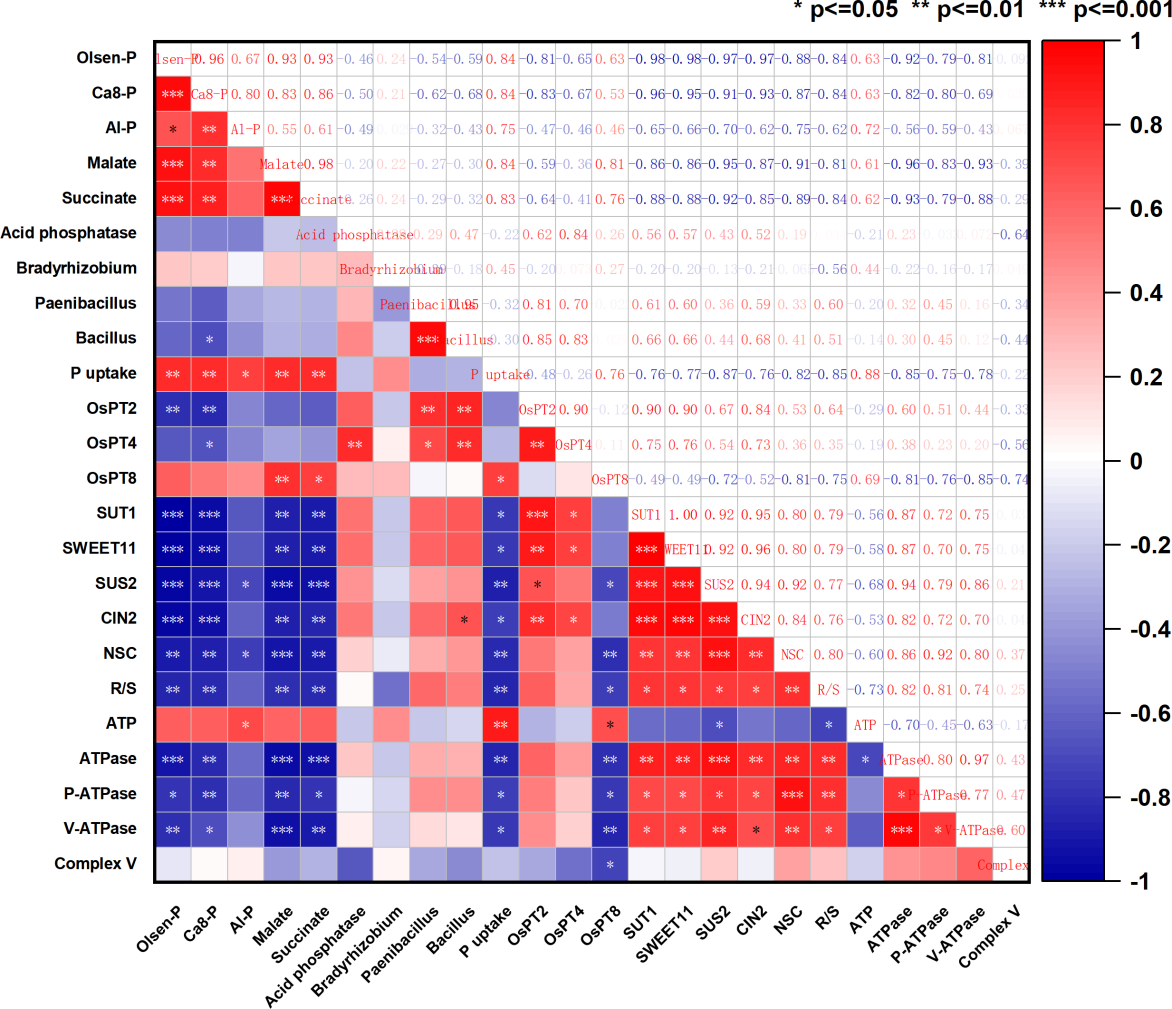
Correlation analysis.

Plant growth relies on water and mineral nutrients, and the RSA is crucial for obtaining these resources from the soil (Hans et al., 2006). The morphology and distribution of root systems play a crucial role in phosphorus uptake and vary with changes in soil phosphorus levels (Liu, 2021). This study found that the addition of silicon did not significantly affect root weight, but it reduced root branching strength and root length density (**Supporting Information**, **Supplementary Figure S4**) and increased root diameter. This suggests that silicon primarily influences root diameter development rather than branching. Shallow roots are beneficial for phosphorus absorption when phosphorus is low, as crops inhibit the elongation of the main root and the growth of root diameter, thereby promoting lateral root development (Sun et al., 2018; Hans et al., 2006). When phosphorus is abundant, crops will inhibit the growth of lateral roots and allocate nutrients for main root elongation and increased root diameter (Lynch, 1995). The author previously found that adding silicon under phosphorus-sufficient conditions can promote root growth and increase root hair quantity in rice under dry cultivation (Jiang et al., 2022, 2023a). This experiment found that adding silicon mainly promotes root volume under low phosphorus conditions. In the case of phosphorus deficiency, the increase in the root-to-shoot ratio is primarily due to the decrease in stem growth and an increase in carbon distribution from stem to root (Hermans et al., 2006). Plant development in this experiment under different phosphorus dosages is consistent with previous conclusions. There is a significant positive correlation between root NSC and root shoot ratio (**Figure 8**). Further investigation revealed that silicon addition promoted leaf area and plant biomass under both 0P and 25P conditions. The addition of silicon significantly promoted aboveground development under the 25P treatment, accompanied by a decrease in the root-to-shoot ratio. The key finding is that the increase in the root-to-shoot ratio under low phosphorus treatment results from enhanced root respiration, which mobilizes NSC transport to the root system, influencing the development of stems and leaves.

### Silicon addition enhances soil phosphorus availability through carboxylate and microbial pathways

There are various forms of phosphorus with different bioavailability in soil (Wei et al., 2020; Liu et al., 2019). In this experiment, the addition of silicon had a greater phosphorus activation effect on the 25P treatment than on the 0P treatment. The decrease in Fe-P after silicon addition may be due to the reduction and dissolution of trivalent iron, which is key to the increase in Olsen-P. Silicon addition simultaneously increases the intermediate cycle phosphorus components (Ca_8_-P and Al-P) as reserves, and Olsen-P and P uptake are significantly positively correlated with Ca_8_-P and Al-P (**Figure 8**). Ca_10_-P and O-P are relatively stable and refractory phosphorus components that are difficult for plants to absorb and utilize (Cao et al., 2020). Excessive phosphorus application leads to the accumulation of significant amounts of excess phosphorus (residual phosphorus) in the soil, which pollutes surface water (Bouwman et al., 2017). We found that adding silicon decreases the content of Ca_10_-P in soil, offering a new approach to addressing phosphorus pollution. This study presents a novel approach to reducing phosphorus application. Farmers may not necessarily need to apply more phosphorus fertilizer when practicing water-saving rice cultivation; however, this does not imply that silicon fertilizer can be used without restrictions. Therefore, further research is required to optimize the nutrient efficiency of silicon-phosphorus interactions.

P deficiency triggers the release of root exudates, including acid phosphatases and RNases (Wang and Liu, 2018), carboxylates, and protons (Wang and Lambers, 2019), which aid in dissolving phosphorus and releasing it from organic phosphorus compounds, thereby increasing phosphorus availability. Organic anions enhance the soil phosphorus effectiveness by competing with both inorganic and organic phosphorus for adsorption sites, promoting mineral dissolution, and stimulating plant growth and microbial growth (Liao et al., 2020). This experiment found that adding silicon promotes the secretion of carboxylate in the roots of rice under dry cultivation, and also leads to an increase in the Mn concentration in the leaves (**Figure 3A**; **Supporting information**, **Supplementary Figure S5**). This aligns with the finding by Yu et al. (2023) that using leaf Mn concentration can reflect the phosphorus promoting effect mediated by rhizosphere organic acids. We further discovered that the phosphorus release mechanism of silicon through the carboxylate pathway varies under different phosphorus conditions. The addition of silicon to the 0P treatment primarily increases the content and proportion of citrate and tartrate, whereas the addition of silicon to the 25P treatment mainly increases the content and proportion of malate and succinate. Correlation analysis showed a significant positive correlation between malate, succinate, Olsen-P, and P uptake (**Figure 8**). The promotion effect of silicon on soil acid phosphatase activity is more significant in the 25P treatment.

Currently, the influence of root exudates on the structure of rhizosphere microbial communities has become one of the research hotspots in plant-soil microorganism interactions (Afridi et al., 2024; Bandopadhyay et al., 2024). We found that compared to the 25P treatment, the community composition in the 25Psi treatment was more diverse. The recruitment effect of silicon on phosphate solubilizing bacteria was most evident in *Bradyrhizobium* bacteria and *Fusarium* and *Penicillium* fungi under the 0P treatment. In the 25P treatment, it was mainly reflected in bacteria *Bradyrhizobium*, *Paenibacillus*, *Bacillus*, and fungi *Fusarium*. This indicates that the increased secretion of organic acid anions from the root system stimulates the formation of beneficial microbial communities capable of activating phosphorus. It is interesting to note that this experiment found a decrease in the abundance of *Actinobacteriota* after adding silicon, as well as a decrease in soil fungal density (**Supporting information**, **Supplementary Figure S6**). Previous studies have shown that organic acids may promote phosphorus uptake through direct mobilization or selection of phosphorus solubilizing microorganisms (Zhang et al., 2023; Bandopadhyay et al., 2024). However, there are multiple phosphorus solubilizing bacteria in the soil that work independently or cross over, and *Actinobacteriota* is just one of them. Silicon addition promotes the secretion of organic acids under low phosphorus conditions in rice under dry cultivation and increases the abundance of phosphorus solubilizing bacteria *Bradyrhizobium*, *Paenibacillus*, and *Bacillus*, thereby increasing available phosphorus. Although silicon alleviates low phosphorus limitation, which may weaken the effect and abundance of *Actinobacteriota*, it does not affect plant regulation of root shoot ratio and energy metabolism to absorb phosphorus from the soil and increase phosphorus content. We found that the hydrolysis of phosphorus by silicon is neither an acid phosphatase process driven by actinomycetes nor an AMF driven process. The potential of silicon addition to promote AMF colonization in roots remains to be explored. This experiment indicates that the hydrolysis of phosphorus by silicon is achieved through the selection of phosphorus-solubilizing microorganisms by organic acids.

### Silicon addition regulates phosphorus absorption and glucose metabolism through energy metabolism

Plants obtain phosphorus from the soil in the form of orthophosphate (H_2_PO_4_^−^) through phosphorus transporters located on the cytoplasmic membrane of their roots (Raghothama and Karthikeyan, 2005). This experiment found that the addition of silicon upregulated the expression levels of phosphorus transporters in the 0P and 25P treatments, significantly increasing the root phosphorus content in the 25P treatment. Plants often adjust their respiration to adapt to stressful conditions such as low temperature (Yu et al., 2020), high temperature (Li et al., 2021), and low light (Li et al., 2023), primarily using energy to sustain respiration rather than growth respiration. This study found that compared to normal phosphorus, the 0P and 25P treatments promote energy production and increase energy consumption, whereas silicon addition reduces energy consumption. This occurs because rice under dry cultivation enhances root respiration and increases the activity of complex V and complex I in response to low phosphorus. The decrease in ATP content during low phosphorus is due to an increase in ATPase, which is primarily activated in response to low phosphorus and helps sustain plant growth. Plasma membrane H^+^-ATPase (P-ATPase) generates energy by hydrolyzing ATP to actively pump H^+^ out of the cell, creating a proton driving force that facilitates the transport of nutrient ions into the cell (Palmgren, 2001). Adding silicon increases soil phosphorus availability, reduces phosphorus uptake and energy consumption, decreases P-ATPase content and V-ATPase activity, reduces energy consumption for sustaining respiration, and allocates more energy to growth and respiration, thereby promoting plant development. Respiration consumes glucose, and when phosphorus is deficient, the aboveground parts of the plant allocate more sucrose to the roots (John and Philip, 2011). Correlation analysis showed that root *SUT1*, *SWEET11*, *SUS2*, *CIN2* were significantly positively correlated with NSC and ATPase (**Figure 8**). We found that compared to the 75P treatment, the 0P and 25P treatments allocated more NSC to the roots. Silicon addition down regulated the sucrose transport genes *SUT1* and *SWEET11* in the 25P treatment, reducing sucrose allocation to roots and promoting aboveground development. The root-to-shoot ratio of NSC significantly decreased (**Figure 6D**). Conversion enzymes promote sucrose metabolism and ATP production, primarily responsible for breaking down sucrose into two monosaccharides (Jiang et al., 2020). The downregulation of the sucrose metabolism gene *SUS2* and invertase gene *CIN2* after silicon addition indirectly proves that a phosphorus rich environment reduces the sugar required for root respiration, aligning with changes in energy. In summary, adding silicon regulates energy allocation, facilitates sugar transport, and balances phosphorus uptake, energy consumption, and plant growth in response to changes in phosphorus environment. We found that adding silicon increased the accumulation of phosphorus in roots, but the absorption and utilization of phosphorus in aboveground parts of the plant, along with their underlying mechanisms, require further experimental verification and research.

## Conclusion

**Figure 9:**
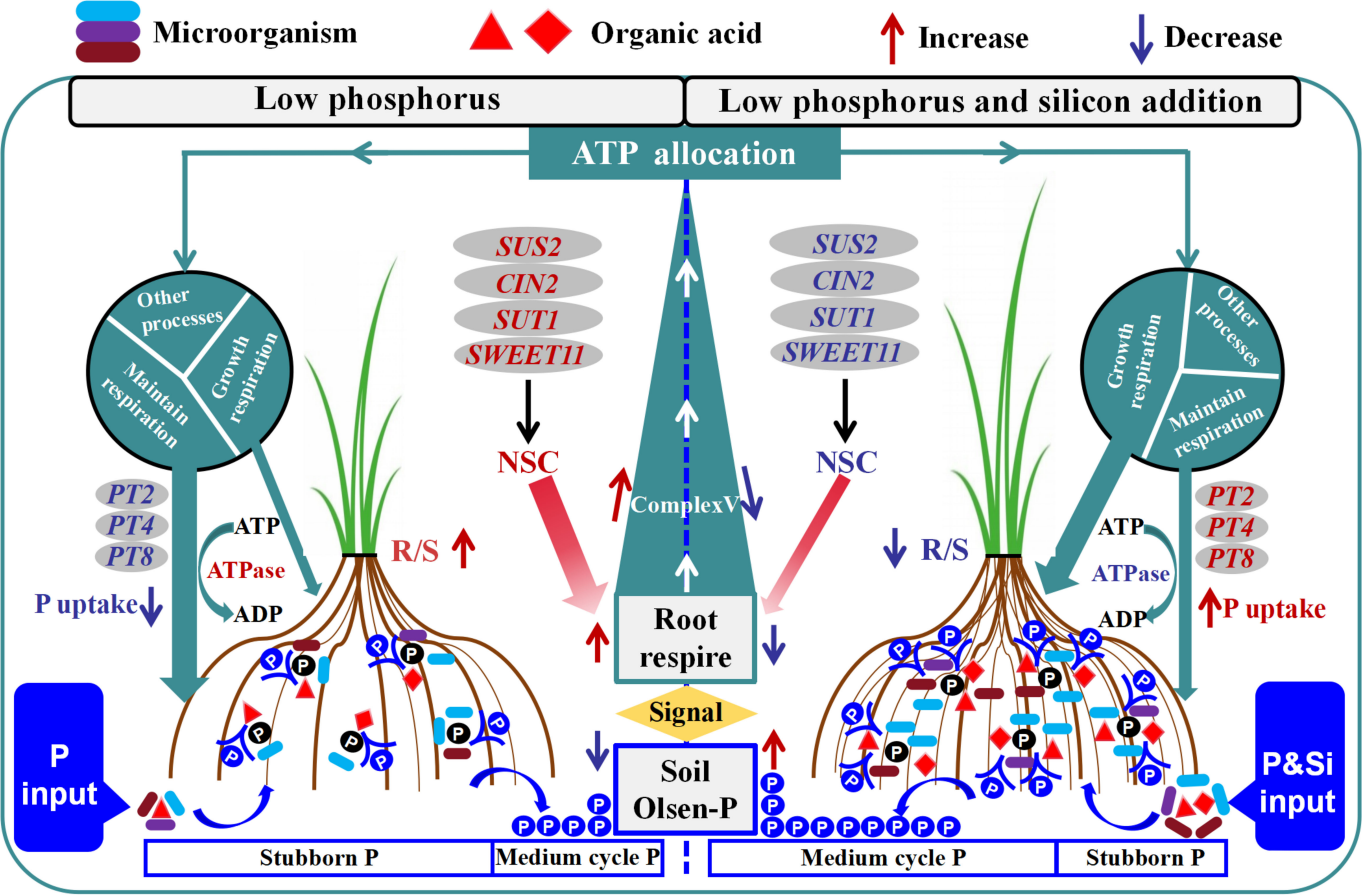
Functional description model of silicon phosphorus interaction in phosphorus conversion and absorption in rice under dry cultivation. (Left) Under low phosphorus conditions, there are fewer root exudates and phosphorus solubilizing microorganisms, with a higher proportion of stubborn phosphorus and less Olsen-P. Root perception of low phosphorus environment enhances respiration and increases Complex V activity. NSC distributes to the roots under the action of *SUT1*, *SWEET11*, *SUS2*, and *CIN2* to supply respiration. Resulting in an increase in R/S. A low phosphorus environment leads to an increase in ATPase, which mainly distributes energy to maintain respiration. Phosphorus transport genes (*OsPT2*, *OsPT4*, *OsPT8*) are downregulated, resulting in lower phosphorus uptake. (Right) After adding low phosphorus and silicon, root exudates recruit phosphorus solubilizing microorganisms to form phosphorus solubilizing groups, increasing the proportion of medium cycle phosphorus and Olsen-P. The activity of Complex V decreased. The increase of NSC in the aboveground part leads to a decrease in R/S. After the improvement of phosphorus environment, ATPase decreases, energy mainly supplies growth respiration, phosphorus transport genes are upregulated, and phosphorus absorption increases. In the figure, red font indicates activation, blue font indicates inhibition. NSC, non-structural carbohydrates; R/S, root/shoot ratio.

We have discovered a crucial production technology—the “silicon-phosphorus partnership” (**Figure 9**)—to enhance phosphorus efficiency and mitigate phosphorus levels in rice under dry cultivation. Silicon addition in low phosphorus conditions can enhance the proportion of circulating phosphorus components in rhizosphere soil, reduce recalcitrant phosphorus components, and thereby increase Olsen-P by 14.37% and root phosphorus content by 7.6%. Silicon addition promotes root volume, phosphatase activity, and carboxylate production, facilitating the targeted recruitment of phosphorus-solubilizing microorganisms. This process improves soil phosphorus availability, and helps regulate energy allocation to maintain a balance between phosphorus uptake and plant growth under fluctuating phosphorus conditions. These findings provide new solutions for efficient cultivation in low phosphorus conditions and enhance our understanding of the role of root-soil-microbe interactions within the silicon-phosphorus partnership in improving the phosphorus environment and regulating phosphorus uptake in rice under dry cultivation. Future research should focus on developing products that integrate silicon-phosphorus composite granulation with phosphorus-solubilizing engineered microbial agents, optimizing nutrient release rates, and exploring the relationship between nutrient release rates of silicon phosphorus partners and nutrient utilization at the plant-root-soil interface.

## Data Availability

The original contributions presented in the study are included in the article/**Supplementary Material**. Further inquiries can be directed to the corresponding author/s.

## Supplementary material

Supplementary Table 1 Design of RT-qPCR reaction primer.

Supplementary Figure 1 Silicon fertilizer information.

Supplementary Figure 2 Plant growth indicators for potted experiment in 2024.

Supplementary Figure 3 Plant growth indicators for field experiments in 2024.

Supplementary Figure 4 Root system architecture of potted plant experiment in 2023.

Supplementary Figure 5 Leaf Mn content.

Supplementary Figure 6 Hyphal abundance.
