# Supplementary figures and images for: Silicon enhanced phosphorus uptake in rice under dry cultivation through root organic acid secretion and energy distribution in low phosphorus conditions
Source: Front Plant Sci. 2025 Mar 24;16:1544893. doi: 10.3389/fpls.2025.1544893 (PMC11973314; doi:10.3389/fpls.2025.1544893)

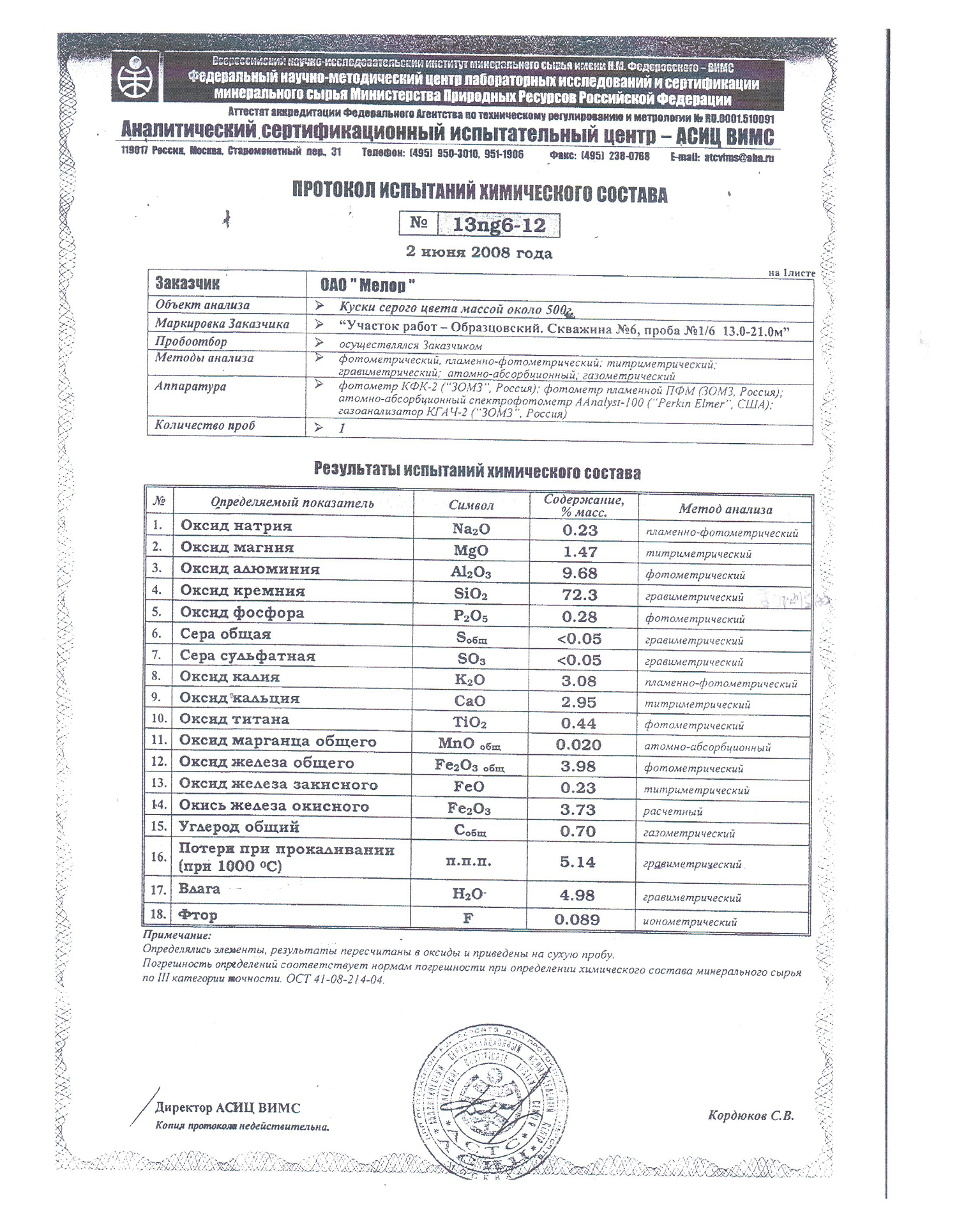

Supplement: Supplementary Figure 1 — Silicon fertilizer information. [file Image1.tif]

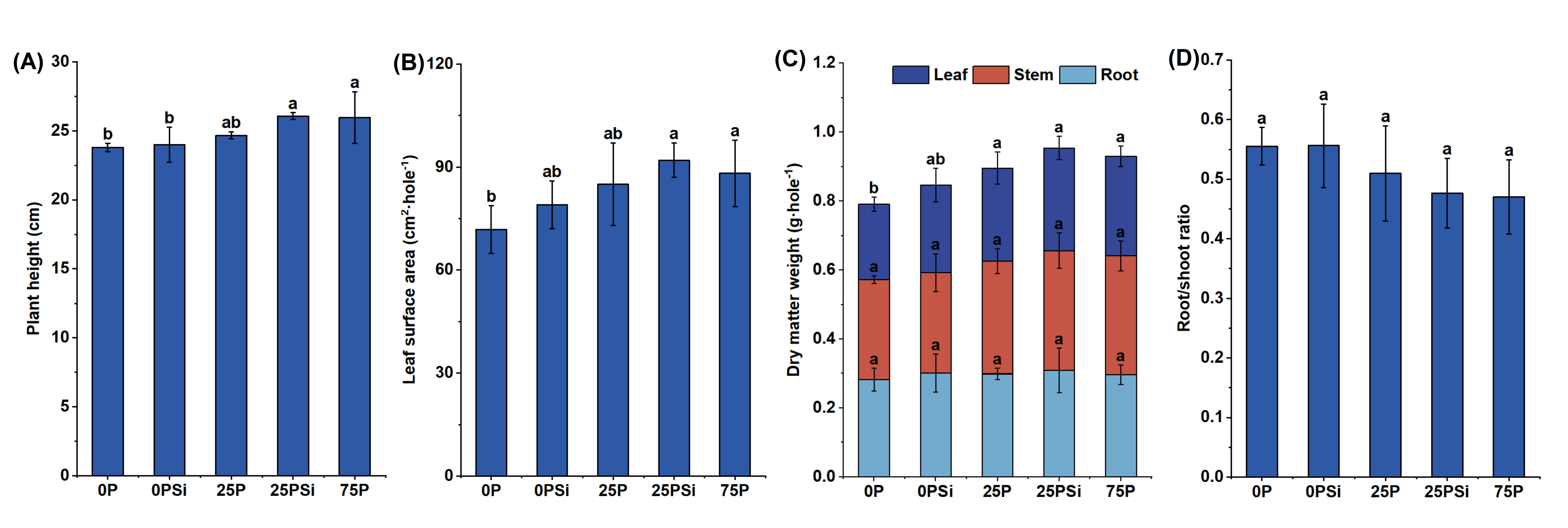

Supplement: Supplementary Figure 2 — Plant growth indicators for potted experiment in 2024. [file Image2.tif]

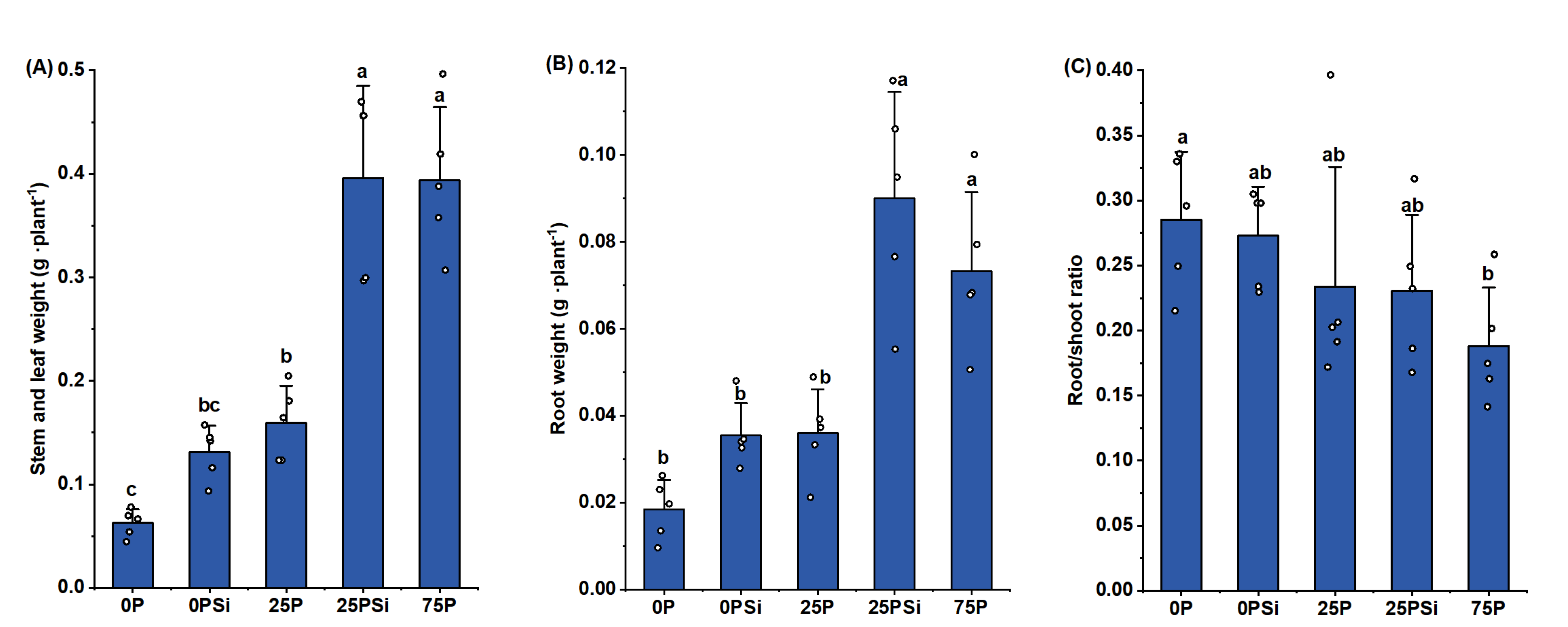

Supplement: Supplementary Figure 3 — Plant growth indicators for field experiments in 2024. [file Image3.tif]

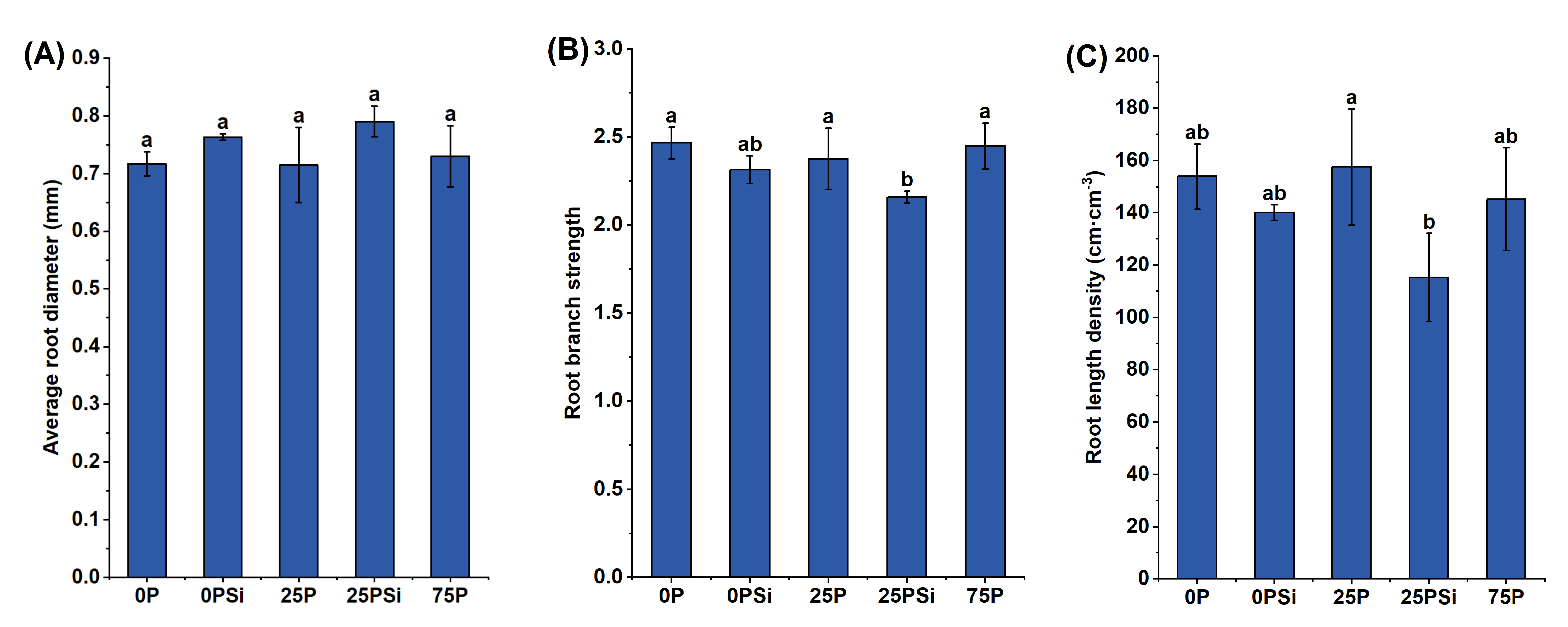

Supplement: Supplementary Figure 4 — Root system architecture of potted plant experiment in 2023. [file Image4.tif]

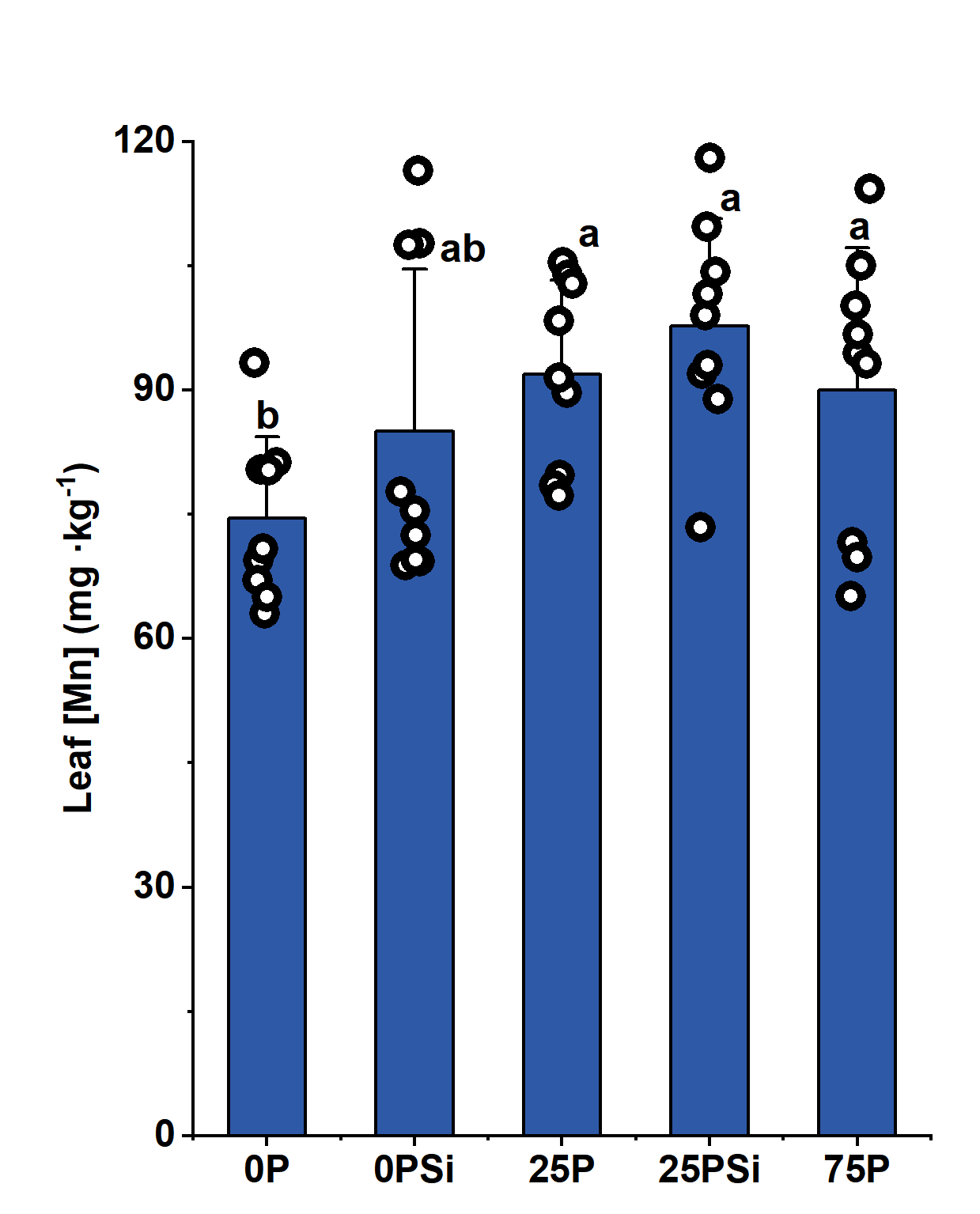

Supplement: Supplementary Figure 5 — Leaf Mn content. [file Image5.tif]

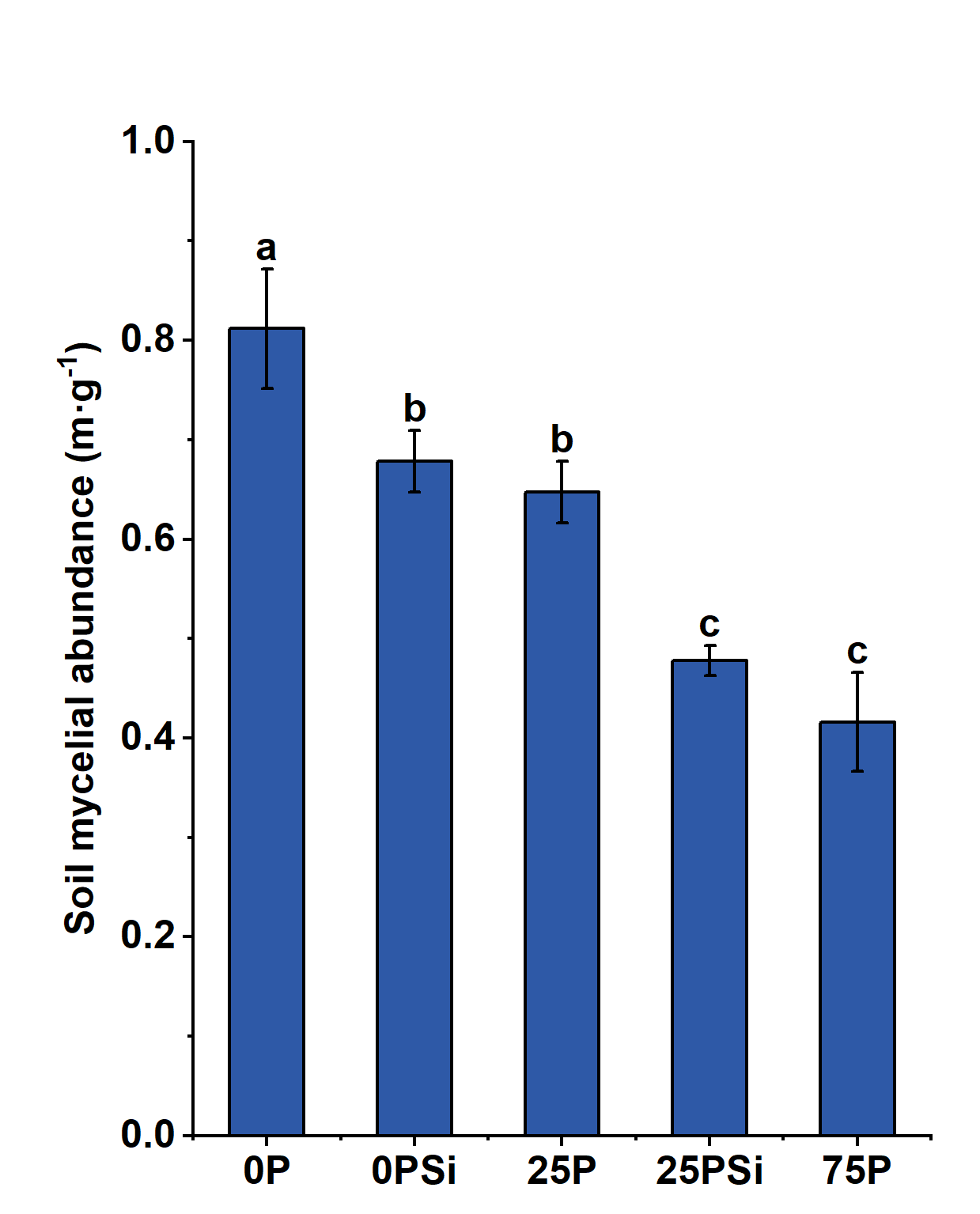

Supplement: Supplementary Figure 6 — Hyphal abundance. [file Image6.tif]
